# Levels of Ca_V_1.2 L-Type Ca^2+^ Channels Peak in the First Two Weeks in Rat Hippocampus Whereas Ca_V_1.3 Channels Steadily Increase through Development

**DOI:** 10.1155/2012/597214

**Published:** 2012-10-14

**Authors:** Audra A. Kramer, Nicholas E. Ingraham, Emily J. Sharpe, Michelle Mynlieff

**Affiliations:** ^1^Department of Biological Sciences, Marquette University, P.O. Box 1881, Milwaukee, WI 53201, USA; ^2^Department of Cell Biology, Neurobiology & Anatomy, Medical College of Wisconsin, 8701 Watertown Plank Road, Milwaukee, WI 53226, USA; ^3^Creighton University School of Medicine, 2500 California Plaza, Omaha, NE 68178, USA

## Abstract

Influx of calcium through voltage-dependent channels regulates processes throughout the nervous system. Specifically, influx through L-type channels plays a variety of roles in early neuronal development and is commonly modulated by G-protein-coupled receptors such as GABA_B_ receptors. Of the four isoforms of L-type channels, only Ca_V_1.2 and Ca_V_1.3 are predominately expressed in the nervous system. Both isoforms are inhibited by the same pharmacological agents, so it has been difficult to determine the role of specific isoforms in physiological processes. In the present study, Western blot analysis and confocal microscopy were utilized to study developmental expression levels and patterns of Ca_V_1.2 and Ca_V_1.3 in the CA1 region of rat hippocampus. Steady-state expression of Ca_V_1.2 predominated during the early neonatal period decreasing by day 12. Steady-state expression of Ca_V_1.3 was low at birth and gradually rose to adult levels by postnatal day 15. In immunohistochemical studies, antibodies against Ca_V_1.2 and Ca_V_1.3 demonstrated the highest intensity of labeling in the proximal dendrites at all ages studied (P1–72). Immunohistochemical studies on one-week-old hippocampi demonstrated significantly more colocalization of GABA_B_ receptors with Ca_V_1.2 than with Ca_V_1.3, suggesting that modulation of L-type calcium current in early development is mediated through Ca_V_1.2 channels.

## 1. Introduction

Calcium is an ideal signaling molecule within neurons because the intracellular concentration is kept very low by calcium binding proteins as well as transporters that sequester calcium in intracellular organelles. Therefore, very small changes in the intracellular calcium concentration can act as a molecular switch, controlling a variety of cellular processes such as regulation of gene expression, neurotransmitter release, propagation of action potentials, synaptic plasticity, neurite outgrowth, cell death, and muscle contraction. Increases in free intracellular calcium can be mediated through release from intracellular stores or by influx through ligand gated or voltage gated channels within the cell membrane. There are 5 broad classes of voltage dependent calcium channels (L, N, P/Q, R, T) characterized by their respective kinetics, voltage dependence, and pharmacological sensitivity (for review, see [1, 2]). The different physiological characteristics of these channels allow for diverse function. In addition to the biophysical properties of the channels, individual channels are located in different regions of neurons reflecting their role in cellular function. For example, the N- and P/Q-type channels are found mainly on the presynaptic terminals of neurons where they control neurotransmitter release [3, 4]. In contrast, L-type calcium channels are typically found on the soma or dendrites of neurons where they regulate enzymatic activity, excitability, and gene expression [5, 6]. Voltage dependent channels are heteromeric structures composed of 4 to 5 different subunits. The *α*_1_ subunit incorporates the conduction pore, the voltage sensor, and gating apparatus, and it is the primary site for regulation by second messengers, drugs, and toxins. Ten distinct *α*_1_ subunits have been isolated by molecular biologists [1, 2]. The auxiliary subunits (*α*_2_ *δ*, *β*, and *γ*) are important in trafficking and modulate the properties of the channels, but the identity is determined by the *α*_1_ subunit. 

Within the different high-voltage classes (L-, N-, P/Q-, and R-type), the L-type calcium channel family (Ca_V_1) characteristically demonstrates long-lasting current and consists of 4 different *α*_1_ isoforms: Ca_V_1.1, Ca_V_1.2, Ca_V_1.3, and Ca_V_1.4. All four L-type channels are sensitive to dihydropyridines but are localized in different tissues and serve different functions in mammals. Ca_V_1.1 is the skeletal muscle L-type calcium channel and is responsible for excitation-contraction coupling [7]. Ca_V_1.4 is primarily located in the retina where it plays a significant role in night vision [8–10]. There is significant overlap in the expression patterns of Ca_V_1.2 and Ca_V_1.3. Ca_V_1.2 is expressed in cardiac and smooth muscle tissue as well as neurons, while Ca_V_1.3 is predominately expressed in neurons. When expressed in *Xenopus *oocytes, these two channel types appear to activate at different potentials and have slightly different sensitivities to dihydropyridines [11]. However, it has not been effectively demonstrated in native neurons that these two properties differ sufficiently to allow for distinction between the two channel types in primary neurons. Therefore, the overlap in expression and lack of pharmacological distinction between these two channel isoforms have hindered the attribution of specific physiological processes to each channel isoform within neurons.

Recent studies using knockout mice have begun to elucidate the functional role of the different L-type channel isoforms in the adult mammalian nervous system (for reviews see [12–14]). For example, conditional Ca_V_1.2 knockouts have been used to determine that Ca_V_1.2, but not Ca_V_1.3 is the main L-type calcium channel that contributes to long-term potentiation in hippocampal cells [15, 16]. Ca_V_1.3 has been found to be the predominate calcium channel isoform that modulates patterns of neural firing in striatal neurons [17]. Ca_V_1.3 knockout mice are deaf due to the lack of calcium currents in both the inner and outer hair cells of the cochlea and have sinoatrial node dysfunction [18–20]. Behavioral experiments have also determined that Ca_V_1.2 activation induces dystonic neurobehavioral syndrome (sustained muscle contractions and self-biting; [21]) while Ca_V_1.3 deficiency can cause antidepressant and anxiolytic-like behavior alone [22].

Only recently have studies begun to address the involvement of different L-type calcium channel isoforms in specific developmental processes using knock-out mice. The auditory system is one area that requires Ca_V_1.3 for proper development [23]. However, calcium influx is likely to be involved in many developmental processes and thus, knocking out the channel throughout the entire developmental period may have profound effects that obscure the attribution of particular responses to specific channel isoforms. It is also possible that in the early developmental period, cells may be able to compensate for the lack of a particular channel isoform by overexpressing a different isoform. Thus, the role of L-type channels in early development has been studied by pharmacological inhibition of the calcium currents. The shift of the GABA_A_ response from excitatory to inhibitory is a major developmental change in neonatal hippocampus in which calcium influx has been implicated. This shift is due to the reversal of GABA_A_ mediated chloride flux that depends on the internal chloride concentration controlled by two chloride transporters NKCC1 (Na^+^K^+^Cl^−^ co-transporter 1) and KCC2 (K^+^Cl^−^ co-transporter 2) [24–28]. Proper maturation of the GABAergic circuitry is dependent on brain-derived neurotrophic factor (BDNF) [29], which has been demonstrated to affect expression of KCC2 [30, 31]. Release of BDNF is mediated by calcium influx through L-type calcium channels, thereby providing one mechanism by which these channels may directly influence hippocampal development. Ganguly and colleagues [26] demonstrated that blockade of calcium influx through L-type calcium channels at the very least, delayed the shift in the reversal potential of chloride currents through GABA_A_ receptors as hippocampal circuits mature. Previous studies in our laboratory have shown that calcium influx through L-type channels is necessary for the upregulation of KCC2 in the first two postnatal weeks in rat hippocampus [32]. In addition, activation of metabotropic GABA_B_ receptors causes facilitation of L-type calcium current primarily through protein kinase C (PKC) activation in these same early neonatal weeks in the hippocampus [29, 33, 34]. There are likely to be many more processes occurring in this early developmental period that are dependent on calcium concentrations. The individual contributions of Ca_V_1.2 and Ca_V_1.3 to these processes are indistinguishable because there are no known pharmacological means of selectively blocking isoforms within the L-type calcium channel family in native neurons. The expression patterns of the calcium channel isoforms may give an indication as to the involvement of these channels in maturation of the nervous system. 

Data from Nuñez and McCarthy [35] demonstrated that expression Ca_V_1.2 peaks between birth and 14 days in the rat hippocampus, suggesting that this L-type channel may be crucial in regulating cellular processes in the early neonatal period. This peak of Ca_V_1.2 expression correlates with the peak of calcium channel enhancement by GABA_B_ receptor activation in the superior region of the hippocampus [32]. However, Park and colleagues [36] demonstrated a protein-protein interaction between the amino terminal of Ca_V_1.3 and the carboxy terminal of the GABA_B_ R2 subunit in adult hippocampus. The present study more closely examines the steady-state expression levels of Ca_V_1.2 and Ca_V_1.3 during the early neonatal period as well as providing an analysis of the localization within the superior region of the hippocampus of the two channel types with the GABA_B_ receptor at one week of age.

## 2. Methods

### 2.1. Animals

All procedures involving animals were approved by the Marquette University Institutional Animal Care and Use Committee according to the guidelines set forth in the National Institute of Health Guide for the Care and Use of Laboratory Animals. All rats were anesthetized briefly with CO_2_ and decapitated prior to brain removal. Sprague-Dawley rats, bred at Marquette University, were utilized for all experiments. 

### 2.2. Western Blot Analysis

The superior region of the hippocampus was isolated from day 1, 4, 6, 8, 12, 15, 21, and 72 rats. Each preparation contained the hippocampi of one to four rats depending on the age and animals were taken randomly from both males and females. Tissue was homogenized in ice-cold buffer (250 mM sucrose, 10 mM Tris, 10 mM HEPES, 1 mM EDTA, pH 7.2 with NaOH) containing fresh protease inhibitors: leupeptin (1 *μ*g/mL), pepstatin (1 *μ*m/mL), and pefabloc (0.5 *μ*g/mL, Sigma-Aldrich, St. Louis, MO). The homogenate was centrifuged at 3,622 X  g for 10 minutes at 4°C. The supernatant was then centrifuged at 49,970 X g for 30 minutes at 4°C. The pellet was resuspended in 100–200 *μ*L of homogenization buffer with protease inhibitors and stored at −80°C. Protein concentrations were determined using a BCA protein assay (Pierce, Rockford, IL) to allow even loading of all lanes. Uniformity of loading was further verified by analyzing bands labeled with actin antibodies (see details below).

For Western blot analysis, NuPage LDS (lithium dodecyl sulfate) sample buffer and NuPage sample reducing agent (Invitrogen, Carlsbad, CA) were added to each preparation prior to a 10-minute incubation at 70°C. Proteins (11 *μ*g per lane) were separated through SDS-polyacrylamide gel electrophoresis using 3–8% Tris-Acetate NuPage Novex 15 lane minigels (1.0 mm; Invitrogen, Carlsbad, CA). Proteins were transferred onto polyvinylidenedifluoride membranes (0.45 *μ*m pore size), which had been soaked in 100% methanol for 3 minutes. To optimize the transfer of large proteins, methanol was omitted from the transfer buffer (384 mM Glycine, 49 mM Tris-base, 0.35 mM sodium dodecyl sulfate, 0.1% NuPage antioxidant). After a 5-minute incubation in phosphate buffered saline (PBS, 134.4 mM NaCl, 4.36 mM KCl, 10.56 mM Na_2_HPO_4_, 1.66 mM NaH_2_PO_4_, pH 7.4 with HCl) the membrane was immersed for 2 hours in 10 mL of blocking buffer (PBS containing 0.05% tween, 5% nonfat dry milk, and 0.1% bovine serum albumin). The membrane was decorated at 4°C overnight with the following primary antibodies: polyclonal rabbit anti-Ca_V_1.2 (1 : 500 dilution, Ca^2+^ CP *α*1C (N-17)-R, sc-16229-R, lot F1606; Santa Cruz Biotechnology, Inc., Santa Cruz, CA), polyclonal rabbit anti-Ca_V_1.3 (1 : 500 dilution, ACC-005; Alomone Labs Ltd., Jerusalem, Israel), and polyclonal rabbit anti-*β*-actin (1 : 1000 dilution; Cell Signaling Technology, Danvers, MA) dissolved in blocking buffer. Membranes were incubated for 1.5 hours at room temperature with goat anti-rabbit HRP conjugated secondary antibodies (1 : 5000 dilution in blocking buffer; Pierce, Rockford, IL) before the addition of Super Signal West Dura Extended Duration substrate (Pierce, Rockford, IL) for visualization via enhanced chemiluminescence using Classic Autoradiography Blue Film BX (Midsci, St. Louis, MO).

Bands were prepared for quantification by subtraction of the background intensity using ImageJ software (developed at U.S. National Institutes of Health and available at http://rsb.info.nih.gov/ij/). The integrated optical densities (IOD) of Ca_V_1.2/Ca_V_1.3 and *β*-actin bands were obtained using Labworks 4.6 imaging and analysis software (UVP, Upland, CA). The Ca_V_ IOD was divided by the *β*-actin IOD measurements to provide an additional control for loading beyond the BCA assay. Both calcium channel antibodies labeled two bands on the Western blots. For Ca_V_1.2, the bands were analyzed individually since they appeared to have different developmental expression patterns. For Ca_V_1.3, the IODs of the bands were added together since their expression pattern through development appeared similar. Each experiment was performed on 3 or 4 different protein preparations for each time point to allow for statistical analysis.

### 2.3. Confocal Microscopy

Hippocampi from day 1, 4, 6, 8, 12, 15, 21, and adult rats were dissected and fixed in a solution of 4% paraformaldehyde (Sigma-Aldrich, Saint Louis, MO) in PBS (defined above) for 30 minutes followed by cryoprotection in 30% sucrose in PBS for 90 minutes. The tissue was embedded in optimal cutting temperature mounting medium (OCT, Sakura Finetek, Torrance, CA) and frozen on dry ice. Transverse hippocampal sections (25 *μ*m) were permeabilized in a solution of PBS with 0.5% Triton X-100 for 20 minutes and blocked for nonspecific binding with 10% goat serum (Invitrogen, Carlsbad, CA) in PBS with 0.05% Triton X-100 for 60 minutes. All antibodies were made in PBS with 0.05% Triton X-100 and 0.1% goat serum. The sections were incubated with either polyclonal rabbit anti-Cav1.2 (1 : 500 dilution, Ca^2+^ CP *α*1C (N-17)-R, sc-16229-R, lot F1606; Santa Cruz Biotechnology, Inc., Santa Cruz, CA) or polyclonal rabbit anti-Cav1.3 (1 : 200 dilution, Ca^2+^ CP *α*1D (H-240), sc-25687, lot B2208) for 2 hours and then were washed 3 times in PBS. Colocalization of L-type calcium channels and GABA_B_ receptors was carried out in tissue obtained from 6–8 day old rats by mixing monoclonal mouse anti-GABA_B_R1 (1 : 500 dilution, Neuromab, UC Davis, CA) with the calcium channel antibodies prior to the initial incubation. Control sections were processed in parallel by omitting the primary antibodies from the PBS with Triton X-100 and goat serum. Tissues were incubated with secondary antibodies, Dylight 549-conjugated Affinipure Goat Anti-Rabbit IgG (1 : 1000 dilution; Thermo Scientific, Rockford, Il), and Dylight 488-conjugated Goat Anti-Rabbit IgG (1 : 500 dilution; Thermo Scientific, Rockford, Il) for 1 hour and washed 3 times in PBS. For the colocalization studies, a mixture of Dylight 549-conjugated Affinipure Goat Anti-Mouse IgG (1 : 1000 dilution; Thermo Scientific, Rockford, Il) and Dylight 488-conjugated Goat Anti-Rabbit IgG (1 : 500 dilution; Thermo Scientific, Rockford, Il) was used to label the respective primary antibodies. Mounting medium with DAPI (4,6-diamidino-2-phenylindole; Vector Laboratories, Burlingame, CA) was applied and slides were covered with a coverslip and sealed. The sections were imaged using a Nikon Perfect Focus Ti-E confocal microscope and NIS Elements imaging software (Nikon Instruments, Melville, NY). 

To analyze the calcium channel distribution over time, images were obtained from 3–9 different rats for each time point studied. Since fluorescence intensity may differ in different preparations processed on different days, no direct comparison of total staining intensity in the CA1 region of the hippocampus was made across preparations and ages. However, analysis was performed by normalizing the intensity of staining within a single section in the different regions (distal radiatum, proximal radiatum, pyramidal cell layer, and stratum oriens). The intensity of the distal radiatum, proximal radiatum, and stratum oriens were normalized by dividing by the intensity of the pyramidal cell layer in an individual section. A two-way ANOVA followed by multiple comparisons using the Holm-Sidak method (Sigmastat 3.5) allowed for analysis of the different regions at the ages studied. 

To analyze the colocalization of Ca_V_1.2 and Ca_V_1.3 L-type channel isoforms with GABA_B_ receptors regions of interest (ROI's) were drawn to encompass the entire stratum oriens, the pyramidal cell layer, the stratum radiatum, and the area of highest visible overlap between the fluorophores on each image. Using the NIS Elements imaging software, the Pearson correlation coefficient (PCC) was determined for each of the regions to quantify the degree of colocalization between Ca_V_1.2 and Ca_V_1.3 L-type calcium channel isoforms, and GABA_B_ receptors in the neonatal rat hippocampus. The PCC is a measure of the overlap of the pixels for the two fluorophores. The PCC was obtained for each ROI, and the values for three different rats were averaged for each region. The higher the PCC, the more probable it is that the two proteins are overlapping in that region of the section. 

## 3. Results

### 3.1. Analysis of Steady-State Expression during Development

**Figure 1:**
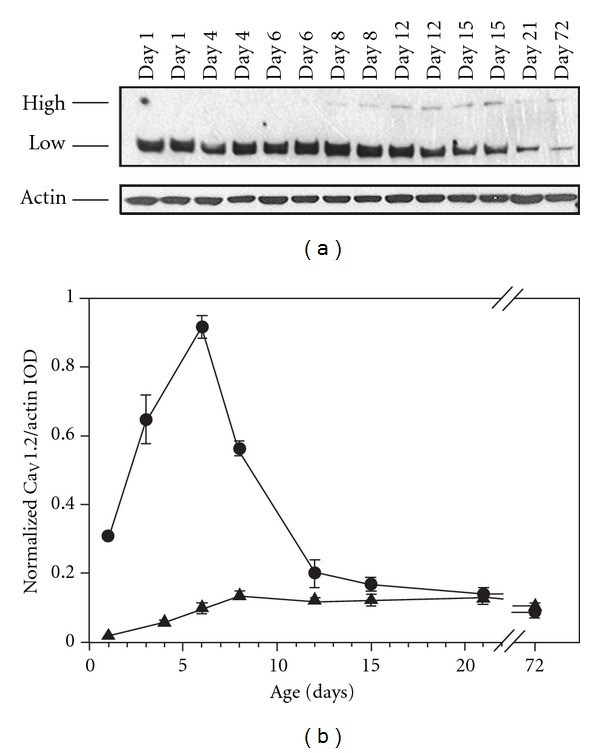
Steady-state expression of L-type Ca_V_1.2 channels in the superior region of rat hippocampus. (a) Representative Western blot analysis of proteins extracted from the superior region of hippocampi obtained from postnatal day 1 through 72 rats. Santa Cruz polyclonal anti-L-type Ca^2+^ CP *α*1C (N-17)-R antibodies labeled a high molecular weight band of ~240 kDa and a low molecular weight band of ~170 kDa (top panel). Antibodies against *β*-actin were used as loading controls for each lane and labeled a band of ~45 kDa (bottom panel). (b) L-type Ca_V_1.2 channel steady-state expression was determined using the integrated optical density (IOD) of each band, which was divided by the IOD of the band labeled with *β*-actin antibodies to account for variations in loading. The ratio of IOD/actin for each preparation was further normalized by dividing by the highest ratio from the same film. The triangles (▴) represent the high molecular weight band and the circles (*⚫*) represent the low molecular weight band. Data represent mean ± sem (*N* = 3 or 4).

Two bands of ~170 kDa and ~240 kDa were labeled with the primary antibodies against Ca_V_1.2 (Figure 1(a)). The specificity of the antibodies used against Ca_V_1.2 was previously determined by Huster et al. [37] in smooth muscle *α*_1_C-subunit calcium channel knockout (SMACKO) mice. The low-molecular-weight band was the most prominent with the antibody lot used for the experiments described in this study. A different lot of antibodies from Santa Cruz only labeled the high-molecular-weight band. This band was faint and difficult to detect regardless of the antibody lot utilized. The high-molecular-weight band was undetectable at postnatal day 1 (P1) and rose steadily until P8 (Figure 1(b)). The low levels of the high-molecular-weight band seen at P8 remained level through adulthood. The low-molecular-weight form was expressed at low levels immediately after birth and rapidly rose to peak levels at P6 (Figure 1(b)). There was a rapid fall off in steady-state expression following this peak, reaching a low level of steady-state expression by P12 that persisted through adulthood. 

**Figure 2:**
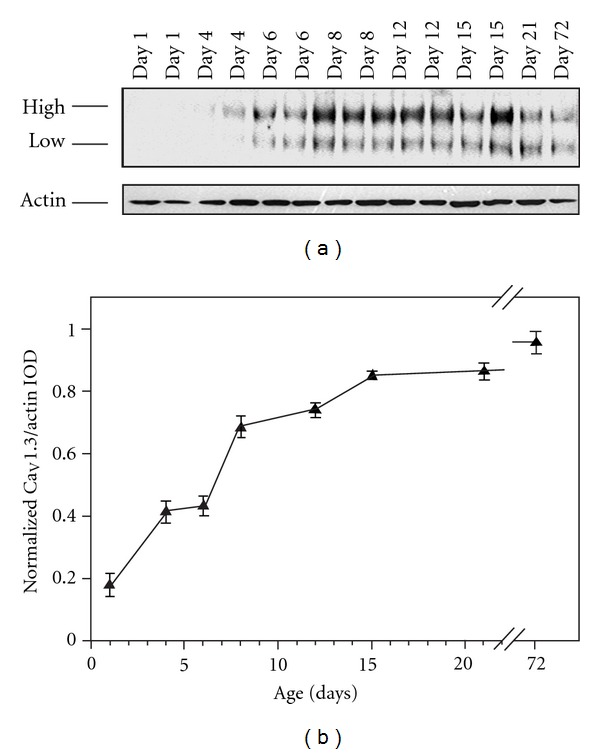
Steady-state expression of L-type Ca_V_1.3 channels in the superior region of rat hippocampus. (a) Representative Western blot analysis of proteins extracted from the superior region of hippocampi obtained from postnatal day 1 through 72 rats. Alomone Labs Ltd polyclonal anti-L-type Ca_V_1.3 (ACC-0050) antibodies labeled a high molecular weight band of ~230 kDa and a low molecular weight band of ~200 kDa (top panel). Antibodies against *β*-actin were used as loading controls for each lane and labeled a band of ~45 kDa (bottom panel). (b) L-type Ca_V_1.3 channel steady-state expression was determined using the integrated optical density (IOD) of the two bands added together, which was divided by the IOD of the band labeled with *β*-actin antibodies to account for variations in loading. The ratio of IOD/actin for each preparation was further normalized by dividing by the highest ratio from the same film. Data represent mean ± sem (*N* = 3 or 4).

Antibodies against Ca_V_1.3 labeled two bands of ~200 kDa and ~230 kDa on the Western blots (Figure 2(a)). The specificity of the antibodies used against Ca_V_1.3 was previously determined by Fossat et al. [38] by probing the spinal cord for Ca_V_1.3 expression following channel knockdown using a peptide nucleic acid- (transportan 10-PNA conjugates) based antisense strategy. In contrast to the Ca_V_1.2 bands, these two bands were relatively even in their expression over time with neither dominating significantly over the other. Thus, the integrated optical density of both bands were added together for the final analysis of steady-state expression (Figure 2(b)). The steady-state expression level of Ca_V_1.3 was very low on P1 and rose steadily to reach approximate adult levels of expression by P15. 

### 3.2. Localization of Ca_V_1.2 and 1.3 Utilizing Confocal Microscopy

**Figure 3:**
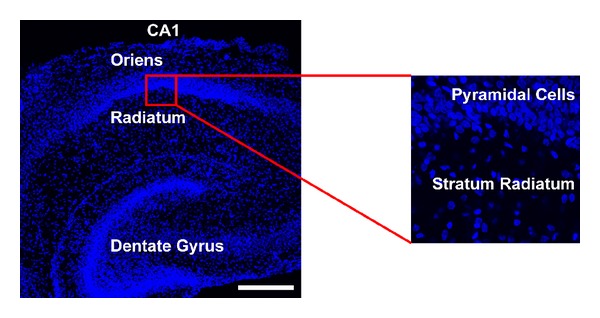
Localization of the CA1 region within the hippocampus for analysis of L-type channel expression. The CA1 region was localized using a low-magnification image of DAPI-stained nuclei (blue) shown on the left. The stratum oriens, stratum radiatum, and dentate gyrus are labeled in this low magnification image and the white scale bar represents 100 *μ*m. The panel on the right illustrates the high magnification image utilized for the analysis of Ca_V_1.2 and Ca_V_1.3 channel density with the stratum radiatum and the pyramidal cell layer labeled.

In order to distinguish between the different hippocampal regions stained with antibodies against Ca_V_1.2 and Ca_V_1.3, all of the sections were stained with the nuclear stain DAPI (4′,6-diamidino-2-phenylindole) to allow visualization of the cell layers within the hippocampus. Figure 3 demonstrates the various regions of the hippocampus (CA1, dentate gyrus, etc.) using a low-magnification image of a hippocampal section taken from a P8 rat. High-magnification images of the CA1 region including the pyramidal cell layer with either the stratum oriens or stratum radiatum were utilized to analyze the distribution of the channels across the layers of the superior hippocampus. Since sections processed on different days may differ in intensity due to bleaching of fluorophores or slight variations in protocol, we chose not to determine changes in expression by simply measuring fluorescence intensity. However, intensity differences within a single section were analyzed and controlled in the following way to allow comparisons across sections. In order to analyze the expression in different layers, the intensity of each layer was divided by the intensity in the pyramidal cell layer for that particular section. Thus, the expression of the channels in each layer is conveyed as a ratio measurement, always compared to the pyramidal layer of that particular section. The trend of localization remained similar within a specific age group, but the absolute fluorescence intensity of a section varied on different days of staining. The expression was measured in the distal region of the stratum radiatum (~100 *μ*m from the pyramidal cell layer), the proximal region of the stratum radiatum (immediately adjacent to the pyramidal cell layer), the pyramidal cell layer, and the stratum oriens. The data were analyzed by a two-way ANOVA followed by the Holm-Sidak method of pairwise comparison to investigate differences across ages and across layers of the hippocampus. 

**Figure 4:**
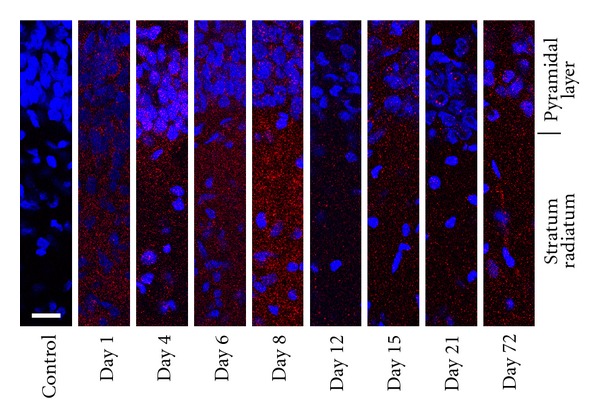
Immunolabeling of Ca_V_1.2 in hippocampus isolated from rats of different ages. Antibodies against Ca_V_1.2 demonstrated diffuse labeling throughout the layers of the CA1 region of the hippocampus across all ages (day 1, 4, 6, 8, 12, 15, 21, and 72). The far left panel is a representative control slide where the primary antibody has been omitted from the incubation. The white bar on this panel represents 20 *μ*m. Each image was background subtracted using the background intensity of its corresponding control slide. For all images the gamma value was maintained at 1.0 and the same peak intensity value for red was utilized for all the images to facilitate direct comparisons of the images.

**Figure 5:**
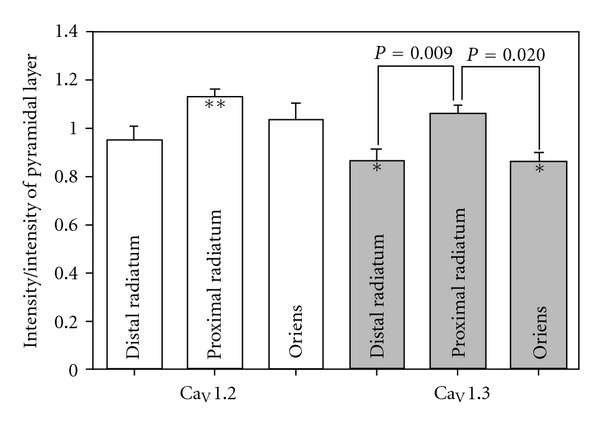
Intensity of staining with Ca_V_1.2 and Ca_V_1.3 antibodies in the CA1 region of the hippocampus. No differences in the distribution pattern of the two channels were noted across the ages tested (day 1, 4, 6, 8, 12, 15, 21, and 72; two-way ANOVA followed by pairwise comparison using the Holm-Sidak method) so that the intensity in each layer was pooled across all ages. The intensity for each layer of every section was normalized by dividing by the intensity in the pyramidal cell layer of that specific section. Comparisons were made between the distal radiatum (~100 *μ*m from pyramidal layer), proximal radiatum, and stratum oriens for each antibody with a two-way ANOVA followed by pairwise comparisons using the Holm-Sidak method. The intensity of the labeling with Ca_V_1.3 antibodies in the distal radiatum and stratum oriens compared to the proximal radiatum was significantly different (unadjusted *P* = 0.009 and *P* = 0.020, resp.). A one sample *t*-test was utilized to compare each layer to the pyramidal layer; **Ca_V_1.2, *P* < 0.001  (*N* = 48); *Ca_V_1.3 *P* < 0.005  (*N* = 43).

**Figure 6:**
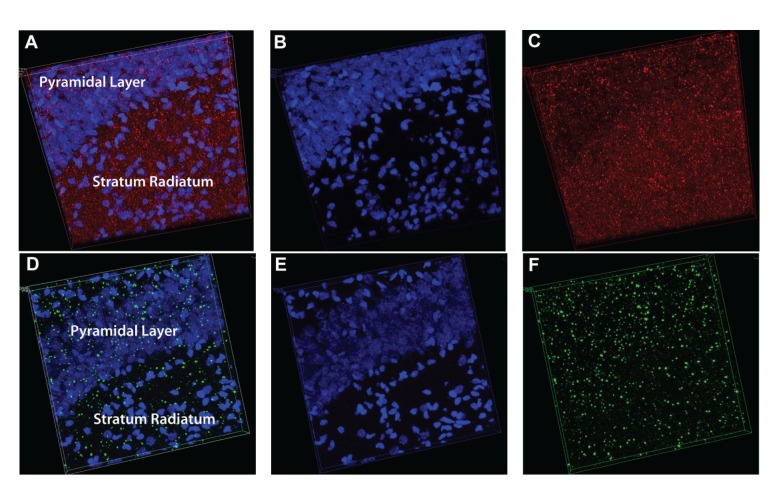
Three dimensional images of Ca_V_1.2 and Ca_V_1.3 antibody labeling in the hippocampal CA1 region. 25 *μ*m sections were imaged as a Z stack of 0.5 *μ*m sections to produce a three dimensional image of labeling with Ca_V_1.2 (A, B, and C) and Ca_V_1.3 (D, E, and F) antibodies. The left panel demonstrates the DAPI stain of the nuclei along with the calcium channel staining with the image split into just nuclei in the middle panel or just calcium channels in the right panel to aid in the visualization of the localization of the channels.

The staining with the Ca_V_1.2 antibody was relatively diffuse throughout the CA1 region of the hippocampus (Figure 4). The intensity of staining appeared greatest around one week of age, corresponding well with the quantification of expression using Western blot analysis. In general, the pattern of expression of Ca_V_1.2 did not change drastically from one age to the next. There was no statistical difference in the distribution of intensity across the cell layers among the different ages and thus, the data from all ages were pooled in Figure 5 (two-way ANOVA followed by pairwise comparison using the Holm-Sidak method). Pooling the data in this manner demonstrated that the expression of Ca_V_1.2 appears higher in the proximal dendritic region (*P* < 0.001, one sample *t*-test, *N* = 48) when compared to the pyramidal cell layer. The expression levels in the distal radiatum and stratum oriens were very similar to the expression in the pyramidal cell layer. This is demonstrated in the 3D reconstruction illustrated in Figure 6(A–C). 

**Figure 7:**
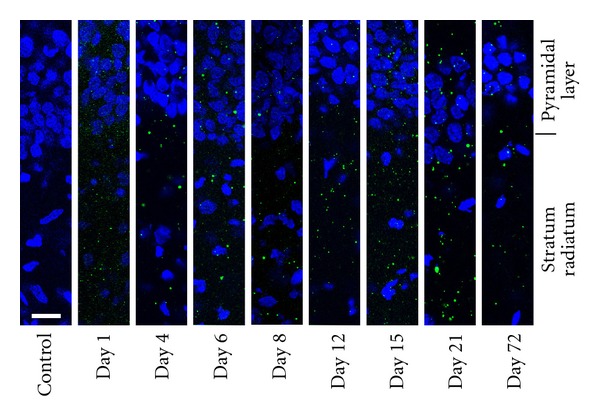
Immunolabeling of Ca_V_1.3 in hippocampus isolated from rats of different ages. Antibodies against Ca_V_1.3 demonstrated punctate labeling throughout the layers of the CA1 region of the hippocampus across all ages (day 1, 4, 6, 8, 12, 15, 21, and 72). The far left panel is a representative control slide where the primary antibody has been omitted from the incubation. The white bar on this panel represents 20 *μ*m. Each image was background subtracted using the background intensity of its corresponding control slide. For all images the gamma value was maintained at 1.0 and the same peak intensity value for green was utilized for all the images to facilitate direct comparisons of the images.

The staining with the Ca_V_1.3 antibody was very punctate with little binding in between intense areas of labeling (Figure 7). The punctate labeling with antibodies against Ca_V_1.3 did not vary over all the ages tested. Labeling with antibodies against Ca_V_1.2 did appear more punctate in older tissue but there still remained diffuse staining throughout the section as well. As with Ca_V_1.2, there was no difference in the distribution of Ca_V_1.3 channels when comparing across ages, but there was a significantly higher amount of labeling in the proximal dendrites when compared to the distal dendrites or the stratum oriens (two-way ANOVA followed by pairwise comparison using the Holm-Sidak method, unadjusted *P* values = 0.009 and 0.020, resp.; Figure 5). The labeling in the proximal radiatum was very similar to that of the pyramidal cell layer but the labeling in the distal radiatum and the stratum oriens was less than the pyramidal cell layer (one sample *t*-test, *P* < 0.005, *N* = 43; Figures 5, 6, and 7). 

### 3.3. Colocalization of Ca_V_1.2, Ca_V_1.3, and GABA_B_ Receptors

**Figure 8:**
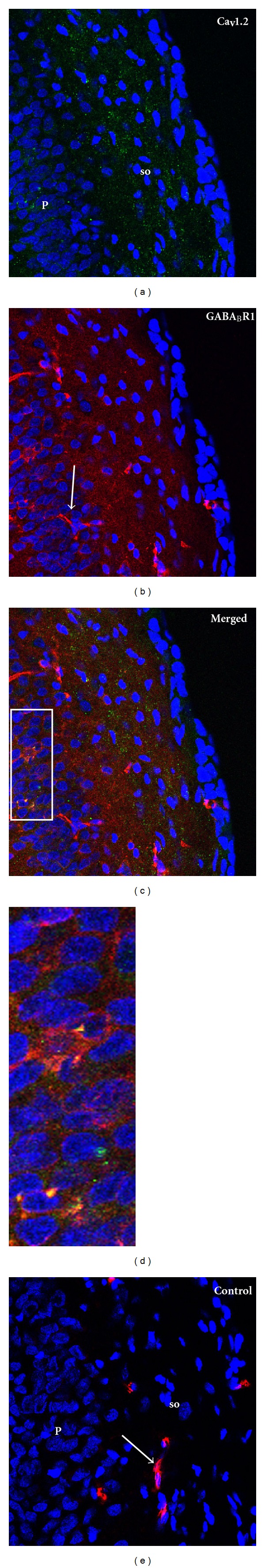
Immunolabeling of CaV1.2 in the CA1 region of rat hippocampus from a 8 day old rat. In each image DNA in the nuclei are stained with DAPI (blue). (a) L-type calcium channel isoform Ca_V_1.2 appears green (Dylight 488). Antibodies against Ca_V_1.2 demonstrate diffuse staining, with only small areas of punctate binding. SO and P indicate locations of the stratum oriens and pyramidal cell layer, respectively. (b) GABA_B_ receptors appear red (Dylight 549). We believe the brightly labeled structures (arrow) represent autofluorescence from blood vessels as they are also present in the control panel E. (c) The yellow staining indicates colocalization of Ca_V_1.2 and GABA_B_ in the first postnatal week in the hippocampus. (d) Higher magnification image from panel C. (e) Control section (omission of primary antibodies) of the stratum oriens. There was some nonspecific staining of blood vessels (red).

**Figure 9:**
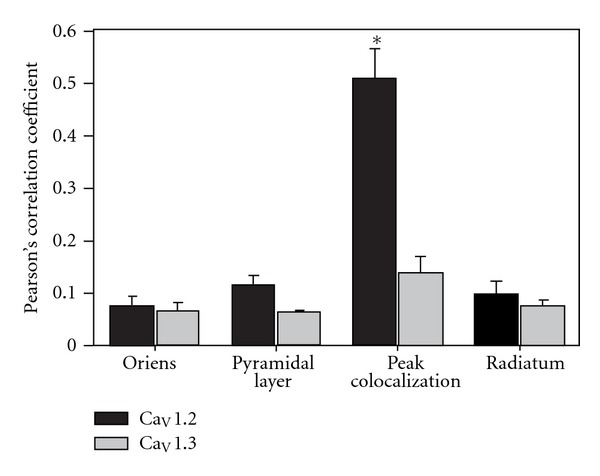
Analysis of calcium channel overlap and GABAB receptors within different areas of the CA1 region of the hippocampus. Regions of interest (ROI's) were drawn on each image to encompass the entire stratum oriens, the pyramidal cell layer, the stratum radiatum, and the area of highest visible overlap between the fluorophores. The Pearson correlation coefficient was obtained for each ROI and the values for three different rats for all of the regions were averaged. The overlap of Ca_V_1.2 with GABA_B_ was significantly higher in individual cells in the pyramidal cell layer than any other region as well as significantly higher than any overlap seen with Ca_V_1.3 and GABA_B_ receptors (two-way ANOVA followed by Student-Newman-Keuls pairwise comparison, *P < 0.001).

As previously seen with antibodies against the two L-type channel isoforms, antibodies against GABA_B_ receptors demonstrated labeling on all three areas of the CA1 region analyzed (Figure 8). Although occasional overlap of the red secondary fluorescent antibody against calcium channel primary antibodies and the green secondary fluorescent antibody against GABA_B_R1 antibodies was seen throughout the sections with Ca_V_1.3 antibodies, it was very sparse. In contrast, there was significant overlap seen with antibodies against Ca_V_1.2 and GABA_B_ receptors throughout the CA1 region of the neonatal hippocampus (Figure 8). The highest areas of overlap were demonstrated in the pyramidal cell layer and often appeared over the whole soma surface. Sections were analyzed for colocalization between the two isoforms of L-type calcium channels and GABA_B_ receptors by determining the Pearson Correlation Coefficient in various regions. The colocalization of Ca_V_1.2 with GABA_B_ was significantly higher in individual cells in the pyramidal cell layer than any other region as well as significantly higher than any overlap seen with Ca_V_1.3 and GABA_B_ receptors (Figure 9; Two-way ANOVA followed by Student-Newman-Keuls pairwise comparison, *P < 0.001). 

## 4. Discussion

The presence of two bands on the Western blots corresponds to reports of a long and short form of Ca_V_1.2 in rats. Typically the short isoform has been termed neuronal while the long form has been described as the cardiac isoform [39–42], which correlates well with our data demonstrating a much higher expression level of the low-molecular-weight band. However, there is also the higher-molecular-weight band present at low levels, which is likely to correspond to the cardiac form. Shistik et al. [43] demonstrated that the longer form of Ca_V_1.2 is also expressed in rat brain. Recent studies in a variety of tissues, including smooth muscle and neurons, have demonstrated data similar to ours with two bands of varying in size from ~180–240 kDa [44–48]. The precise molecular weight will vary depending on the extent of glycosylation present in the protein preparations. Western blot analysis of Ca_V_1.3 also demonstrated two bands of varying molecular weight. Two splice variants of Ca_V_1.3 have been described in the literature that either use exon 42 or exon 42A to encode a longer or shorter isoform of the channel [49].

Analysis of the developmental expression of Ca_V_1.2 by Western blot correlates well with studies by Nuñez and McCarthy [35] that also utilized Western blot analysis to determine steady-state protein expression. They found significant differences when comparing males versus females in the calcium influx by activation of GABA_A_ receptors in the early neonatal period due to chloride efflux mediated depolarization. However, the differences in depolarization can be explained by the different expression patterns of NKCC1 and KCC2 in males versus females rather than any differences in calcium channel expression. Their data on calcium channel expression demonstrated low expression of Ca_V_1.2 at P0 with a large increase by P7, which decreased by P14 in the hippocampal CA1 region with no significant difference in male and female rats. The data presented in the current study was pooled from both males and females. No developmental studies of Ca_V_1.3 have been performed in hippocampal tissue utilizing Western blot analysis of protein levels. Schlick et al. [50] used RT-PCR analysis of mRNA for Ca_V_1.2 and Ca_V_1.3 in whole hippocampus of mice to study developmental changes in L-type calcium channels. Comparisons were made between hippocampal tissue isolated from embryonic day 18, P0, P14, and P56 mice. It is important to note that while mRNA levels may reflect the final protein expression, it is also possible to have differences in translation or protein degradation rates, which could alter the interpretation of the data. If degradation rates for the protein vary, changes in the mRNA transcript levels would not necessarily directly correlate with changes in protein expression. mRNA levels for Ca_V_1.2 were highest in hippocampi of embryonic and P0 mice. These data correspond well with our protein expression studies since the appearance of mRNA levels should precede the protein expression. mRNA levels for Ca_V_1.3 remained relatively steady at all ages tested. The difference between the protein expression levels reported here for Ca_V_1.3 and the mRNA levels reported by Schlick et al. [50] could be due to differences between mice and rats as well as differences in Ca_V_1.3 protein translation or degradation rates with age. Kim et al. [51] used Western blot analysis to study the developmental expression of Ca_V_1.3 in the rat central nervous system and the data are very similar to the data reported here. 

Confocal analysis of the antibody labeling intensity demonstrated variations in the distribution of the channels across the layers of the CA1 region of the hippocampus, but the relative distribution between layers remained the same throughout development for both Ca_V_1.2 and Ca_V_1.3. The data presented here are in agreement with Hell and colleagues [5, 6] who demonstrated higher expression of Ca_V_1.2 and Ca_V_1.3 in the proximal dendritic region of the superior hippocampus in adult rat. Hell and colleagues [5, 6] did not characterize the distribution of the channels at younger ages in rat hippocampi. 

The analysis of protein expression by Western blot demonstrates that the Ca_V_1.2 isoform of the L-type calcium channel predominates in the early neonatal period suggesting that this is the channel that contributes to developmental processes, such as the KCC2 upregulation [32]. This also suggests that it is modulation of Ca_V_1.2 that accounts for the enhancement of L-type calcium current in the neonatal hippocampus by GABA_B_ receptor activation [33, 34]. Colocalization of antibodies labeling GABA_B_ receptors was also much more prominent with Ca_V_1.2 antibodies than with Ca_V_1.3 antibodies providing further evidence that GABA_B_ receptors are more likely to modulate Ca_V_1.2 than Ca_V_1.3 channels. Studies by Park et al. demonstrated an interaction between GABA_B_ receptors and Ca_V_1.3 channels [36] in adult hippocampus. More recently, a study from the same laboratory [52] demonstrated that GABA_B_ receptors enhanced calcium influx through both Ca_V_1.2 and Ca_V_1.3 when expressed in HEK293 cells indicating that both channels may potentially interact with GABA_B_ receptors. 

Western blot analysis of protein expression provides a characterization of large changes in protein expression but may not be sensitive enough to detect small changes in protein expression, particularly if the baseline expression is low. Confocal images taken of tissue labeled with antibodies against both Ca_V_1.2 and Ca_V_1.3 demonstrate that although the total protein levels of Ca_V_1.3 were low on the Western blots, there is significant expression of Ca_V_1.3 even in tissue taken from 1-day-old rat pups. The binding of Ca_V_1.3 antibodies was very punctate with little binding in between the clusters. These intense regions make it difficult to analyze the overall intensity since there was very little labeling between clusters. It also complicates the analysis of changes in developmental expression of Ca_V_1.3. In order to obtain a reasonable approximation of the average intensity within a region of the hippocampus, the largest possible region of interest within a layer was analyzed. With more diffuse labeling such as that seen with Ca_V_1.2, this procedure accurately reflected the staining within a region. The lack of labeling between the intense punctate staining seen with Ca_V_1.3 causes the intensities measured in a large region of interest to be very low and not necessarily representative of the intensity of staining in the very dense regions. Thus, we cannot rule out that significant amounts of calcium are flowing into the cell through Ca_V_1.3 at highly specific regions, even at the earliest time points tested. It is also possible that, while the colocalization of Ca_V_1.2 and GABA_B_ receptors was more visible, there may also be regulation of Ca_V_1.3 by GABA_B_ receptors as demonstrated by Rhim and colleagues [36, 52]. A direct correlation between studies in our laboratory demonstrating L-type current enhancement is difficult because our studies were performed in acute neonatal hippocampal cultures while the Rhim laboratory primarily used a heterologous expression system (HEK293). 

In conclusion, immunohistochemical analysis with confocal microscopy demonstrated that both Ca_V_1.2 and Ca_V_1.3 L-type calcium channels are expressed from P1 through adulthood in the CA1 region of the hippocampus with the highest concentration in the proximal dendritic region. Quantification of the expression levels by Western blot analysis demonstrated that expression of Ca_V_1.2 peaks around day 8, while expression of Ca_V_1.3 starts low and gradually reaches adult levels by P15. In addition, the highest colocalization between L-type calcium channels and GABA_B_ receptors was found with Ca_V_1.2 in the pyramidal cell layer.
